# Dual-Viral Transduction Utilizing Highly Efficient Retrograde Lentivirus Improves Labeling of Long Propriospinal Neurons

**DOI:** 10.3389/fnana.2021.635921

**Published:** 2021-03-22

**Authors:** Brandon L. Brown, Rachel M. Zalla, Courtney T. Shepard, Russell M. Howard, Jonathan A. Kopechek, David S. K. Magnuson, Scott R. Whittemore

**Affiliations:** ^1^Interdisciplinary Program in Translational Neuroscience, University of Louisville, Louisville, KY, United States; ^2^Kentucky Spinal Cord Injury Research Center, University of Louisville, Louisville, KY, United States; ^3^Department of Anatomical Sciences and Neurobiology, School of Medicine, University of Louisville, Louisville, KY, United States; ^4^Department of Bioengineering, J.B. Speed School of Engineering, University of Louisville, Louisville, KY, United States; ^5^Department of Neurological Surgery, School of Medicine, University of Louisville, Louisville, KY, United States

**Keywords:** spinal cord, adeno associated virus, lentiviral vector, propriospinal, tract-tracing, MATLAB, quantification

## Abstract

The nervous system coordinates pathways and circuits to process sensory information and govern motor behaviors. Mapping these pathways is important to further understand the connectivity throughout the nervous system and is vital for developing treatments for neuronal diseases and disorders. We targeted long ascending propriospinal neurons (LAPNs) in the rat spinal cord utilizing Fluoro-Ruby (FR) [10kD rhodamine dextran amine (RDA)], and two dual-viral systems. Dual-viral tracing utilizing a retrograde adeno-associated virus (retroAAV), which confers robust labeling in the brain, resulted in a small number of LAPNs being labeled, but dual-viral tracing using a highly efficient retrograde (HiRet) lentivirus provided robust labeling similar to FR. Additionally, dual-viral tracing with HiRet lentivirus and tracing with FR may preferentially label different subpopulations of LAPNs. These data demonstrate that dual-viral tracing in the spinal cord employing a HiRet lentivirus provides robust and specific labeling of LAPNs and emphasizes the need to empirically optimize viral systems to target specific neuronal population(s).

## Introduction

Understanding the complexity and specificity of neural pathways and circuits in the mammalian nervous system is a major goal for neuroanatomists and is vital to understand and treat nervous system injuries and disorders (Nassi et al., 2015; Zeng, 2018; Horn and Fox, 2020; Lanciego and Wouterlood, 2020; Ugolini, 2020; Wong et al., 2020). The development and refinement of technologies such as functional near-infrared spectroscopy (Cao et al., 2015; Hu et al., 2020), diffusion weighted magnetic resonance imaging (Jeurissen et al., 2019; Yeh et al., 2020), and resting-state functional magnetic resonance imaging (Fox and Raichle, 2007; Horn and Fox, 2020) have enhanced our understanding of macroscale connectomics, and improved patient treatments and outcomes (Horn et al., 2017; Joutsa et al., 2018; Okromelidze et al., 2020). Mesoscale connectomics–characterizing a single population of neurons and/or connectivity of those neurons–has made similar progress (Lanciego and Wouterlood, 2020; Ugolini, 2020), but traditional tracers such as horseradish peroxidase, cholera toxin subunit B (CTB), hydroxystilbamidine (known commercially as Fluoro-Gold), and conjugated dextran amines [which include biotinylated dextran amine (BDA), and rhodamine conjugated dextran amine (RDA), also known as Fluoro-Ruby (FR) or Mini Ruby depending on molecular weight] remain the most widely used technique. These traditional tracers allow for anterograde and retrograde tract-tracing, are valuable for revealing the locations of neurons projecting to or from and area of interest, and have been widely used throughout the nervous system for more than three decades (Lanciego and Wouterlood, 2011; Wouterlood et al., 2014; Nassi et al., 2015). Despite their extensive use, each of these traditional tracers has limitations including the potential of labeling any/all neurons projecting to or from an area. More specifically, dextran amines can be taken up by axons damaged during injection procedures (Glover et al., 1986), long-term exposure to Fluoro-Gold can be neurotoxic (Naumann et al., 2000), Fluoro-Gold and CTB can inadvertently be taken up by fibers of passage (Dado et al., 1990; Chen and Aston-Jones, 1995), and biotin conjugates of CTB can be transneuronal (Lai et al., 2015), all of which may hinder mesoscale connectomic analyses.

Targeted genetic manipulations and an improved understanding of neurotropic viruses have overcome some of the limitations of traditional tracers and allowed for precise targeting of pathways and evaluation of connectivity throughout the central nervous system (CNS) (Atasoy et al., 2008; Wertz et al., 2015; Deng et al., 2016; Zingg et al., 2017). Since 1998, adeno-associated viruses (AAVs) have been widely used for CNS tract-tracing (Chamberlin et al., 1998). AAVs are neurotropic, permit long-term stable gene expression in neurons, cause little toxicity, and high titer production is easily achieved (Chamberlin et al., 1998; Tervo et al., 2016; Chan et al., 2017). While viral-based tracing using AAVs or other vectors offers advantages over traditional tracers, there are numerous variables that must be understood and optimized if robust and specific tracing is to be achieved. For example, altering the AAV capsid, and therefore the serotype, impacts cellular tropism and changes the volumetric spread at the injection site (Burger et al., 2004; Liu et al., 2005; Hutson et al., 2012; Aschauer et al., 2013) and AAV dosage–volume and titer–influences cellular tropism, transgene expression, and the direction of viral transport (Klein et al., 2002; Hollis et al., 2008; Klaw et al., 2013). Even the purification method and route of delivery can alter AAV transduction (Klein et al., 2008; Rosenberg et al., 2014). Similarly, when using lentiviral vectors for CNS transduction, altering the viral envelope can impact transduction efficiency, immune response, and the direction of viral transport (Kitagawa et al., 2007; Kato et al., 2011a; Hirano et al., 2013; Tanabe et al., 2019).

**FIGURE 1:**
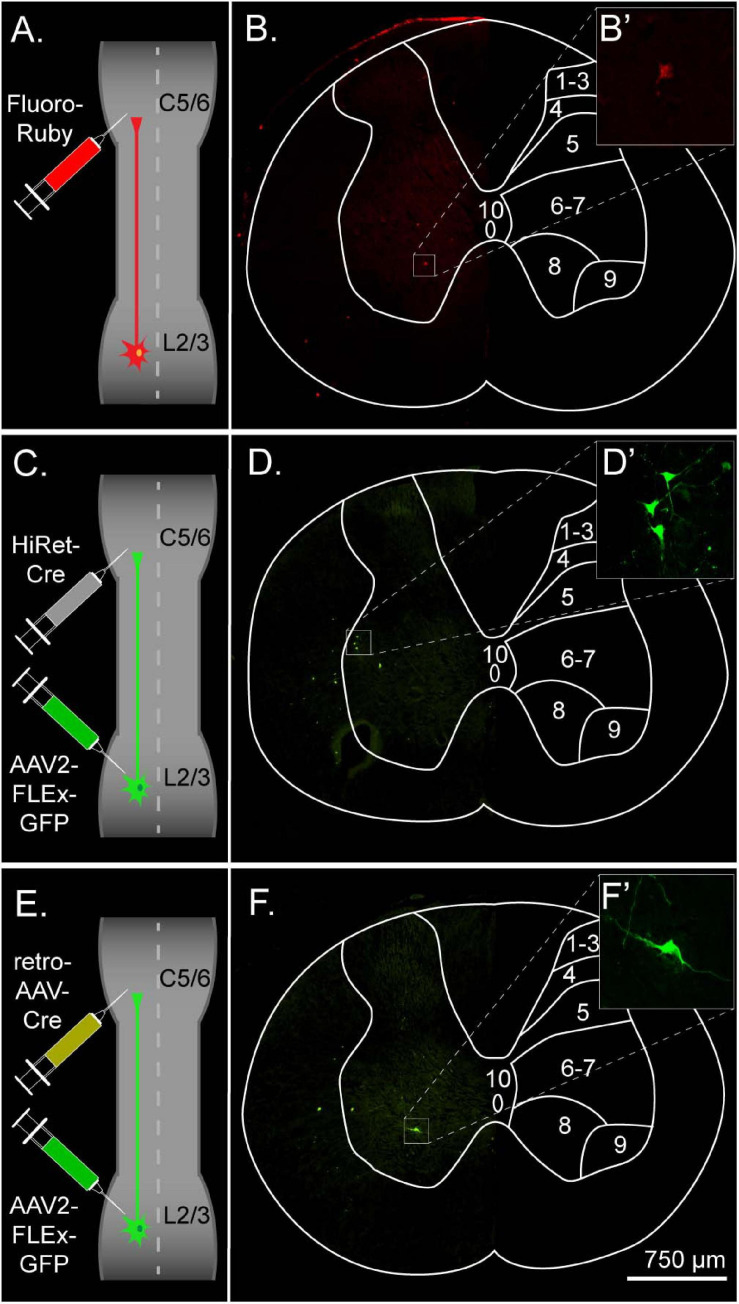
Experimental design and overview for labeling left ipsilateral long ascending propriospinal neurons (LAPNs) in the adult rat. **(A)** The chemical tracer Fluoro-Ruby was injected ipsilaterally at C5–6. **(B)** Lumbar spinal cord cross section showing labeled neurons from Fluoro-Ruby group. **(C)** HiRet-Cre was injected ipsilaterally at C5–6 and AAV2-FLEx-GFP ipsilaterally at L2–3. **(D)** Lumbar spinal cord cross section showing labeled neurons from the HiRet-Cre group. **(E)** RetroAAV-Cre was injected ipsilaterally at C5–6 and AAV2-FLEx-GFP ipsilaterally at L2–3. **(F)** Lumbar spinal cord cross section showing labeled neurons from the retroAAV group. **(B’,D’,F’)** Maximum intensity projections of confocal z-stacks illustrate the intensity and detail of labeled cells.

An improved understanding of viral vector transduction in the CNS, in combination with other technologies such as transgenic labeling and traditional tracers, has led to projects such as the Allen Mouse Brain Connectivity Atlas which has provided a high-resolution map of the mouse brain connectome (Oh et al., 2014). However, viral-based tracing for targeting and mapping the spinal cord has not received as much attention. The main goal of the current study was to evaluate various tracing methods for targeting long ascending propriospinal neurons (LAPNs) in the rat. LAPNs are an ideal system for this as, they have relatively long axons, they have been found in numerous species, and they have similar numbers of ipsi- and contra-lateral projections (Giovanelli Barilari and Kuypers, 1969; Reed et al., 2006; Ruder et al., 2016; Pocratsky et al., 2020). We utilized FR, a 10 kD rhodamine-conjugated dextran amine, as well as two dual-viral systems for target-defined projection labeling (Figures 1A,C,E; Zeng, 2018). AAV2 containing a Cre-dependent flip-excision switch (FLEx) was injected at the level of the cell bodies, lumbar levels 2–3 (L2–3) (Atasoy et al., 2008). For retrograde transduction and Cre delivery, either the retrograde adeno-associated virus (retroAAV) developed by Tervo et al. (2016) or the highly efficient retrograde (HiRet) lentivirus developed by Kato et al. (2011b), was injected at the level of LAPN axon terminals, cervical 5–6 (C5–6) (Figure 1). For either of these vectors to provide efficient retrograde transduction, subsequent FLEx recombination, and labeling of LAPNS, the vector must readily infect axon terminals, undergo retrograde transport, and deliver its cargo to LAPN nuclei.

The process of quantifying labeled neurons in spinal cord tissue sections can be problematic. While the accuracy of manually counting is high, it is inefficient and introduces human error. To improve reproducible counting and reduce the time required for the quantification of large numbers of tissue sections, we developed a custom application using MATLAB (ver. R2019a) to accurately count the number of labeled cells. The application developed allows for easy navigation through large numbers of tissue sections and lets users overlay anatomical maps or grids for more detailed quantification. The application can be downloaded^1^, a step-by-step instructional video shows how to use the application successfully^2^, and details of the program along with written directions can be found in Supplemental Document 1.

## Materials and Methods

### Tracer and Virus Preparation

10% stocks of FR were made by dissolving 10 mg of FR dry powder in 100 μL of distilled water, and 10 μL aliquots were stored at −20°C. FR aliquots were thawed the morning of injection surgeries. 100 μL of AAV2-FLEx-GFP (3.7 × 10^12^ vp/mL) and of retroAAV2-CAG-Cre (5.3 × 10^12^ vg/mL) (referred to here as *retroAAV-Cre*) were ordered from the Gene Therapy Center Vector Core at the University of North Carolina at Chapel Hill. 10 μL aliquots of each were made, stored at −80°C, and thawed the morning of injection surgeries. HiRet-Cre (1.6–2.0 × 10^10^ vg/mL) was produced by Zhigang He’s laboratory (Boston Children’s Hospital) using previously described methods (Wang et al., 2017). 10 μL aliquots of Lenti-HiRet-Cre were stored at −80°C and thawed the morning of injection surgeries.

### Stereotaxic Spinal Cord Injection Surgeries

This animal study was reviewed and approved by the University of Louisville Institutional Animal Care and Use Committee. A total of *N* = 12 female Sprague Dawley rats (200–220 g: Envigo) were evenly divided among the groups (*N* = 4 per group). Prior to surgical procedures, animals were housed two per cage with *ad libitum* food and water under 12 h light/dark cycle. Glass micropipettes for intraspinal injections were pulled from borosilicate glass capillaries (World Precision Instruments, Inc.) using a micropipette puller (Sutter Instrument Co.) and the following parameters: heat = 600, pull = 29, velocity = 57, and time = 150. Pipettes were trimmed to an external diameter of 25 μm, beveled using a micropipette beveller (World Precision Instruments, Inc.), and sterilized with 100% ethanol prior to use. The morning of injection surgeries, individual tracers/viruses were loaded into pipettes and viral pipettes were kept on ice between surgeries to minimize viral degradation.

Animals were anesthetized with a mixture of ketamine, xylazine, and acepromazine (40, 2.5, and 1 mg/kg, i.p.), and supplemented with 1–2.5% isoflurane in 98% oxygen at a rate of 1 L/min as needed. For FR injections, animals were placed into a custom-built spinal stabilization unit (Zhang et al., 2008) and received a C5–6 laminectomy and durotomy to expose spinal levels C5–6. Two, 0.25 μL boluses of FR were injected at same site into the left intermediate gray matter of C5–6 (0.55 mm mediolateral, 1.2 mm dorsoventral) using a stereotaxic device (World Precision Instruments, Inc.) (Reed et al., 2009). Boluses were injected 2 min apart to allow FR to spread throughout the tissue, mitigate extravasation from the injection site, and minimize pressure exerted on the tissue at the injection site. This volume was used as the rostrocaudal spread within the spinal gray matter at the injection site was similar to the rostrocaudal spread of volume of virus(es) injected (data not shown).

For viral injections, animals were placed into the spinal stabilization unit, received a laminectomy and durotomy at thoracic vertebrae 12 to expose spinal L2–3, and a C5–6 laminectomy and durotomy to expose spinal C5–6. Two, unilateral injections of either of HiRet-Cre or retroAAV-Cre were injected into the left intermediate gray matter of C5–6 (0.55 mm mediolateral, 1.2 mm dorsoventral, 1.3 mm rostrocaudal). The two injections were given in two, 0.25 μL boluses with 2 min between injections. AAV2-FLEx-GFP was injected into the intermediate gray matter of L2–3 (0.5 mm mediolateral, 1.35 mm dorsoventral, 1.3 mm rostrocaudal) using the same injection protocol as the C5–6 injections. Following injections, incision sites were sutured in layers and wounds closed with surgical staples. Gentamicin (20 mg/kg) and saline were given subcutaneously prior to animals waking, buprenorphine (10 mg/kg, s.c.) was administered every 12 h for 48 h post-surgery for pain management, and prophylactic doses of gentamicin (20 mg/kg, s.c.) were administered for 7 days. Animals were single housed until surgical staples were removed 7 days post-surgery.

### Tissue Processing, Imaging, and Manual Quantification

Two weeks following FR injections, and 3 weeks following viral injections, animals were anesthetized using a cocktail of ketamine, xylazine, and acepromazine (40, 2.5, and 1 mg/kg, i.p.), and transcardially perfused with phosphate-buffered saline (pH 7.4) followed by 4% paraformaldehyde. Spinal cords were harvested, post-fixed in 4% paraformaldehyde for 1–2 h, and transferred to 30% sucrose for 3–4 days at 4°C. L1–4 spinal segments were isolated, embedded in tissue freezing medium, cryosectioned at 30 μm, slide mounted, and stored at −20°C. For coverslipping, slides were warmed, rinsed in PBS for 5 min, coverslipped with Fluoromount-G (SouthernBiotech), and air dried overnight.

Every other tissue section was imaged to avoid double counting labeled propriospinal neuron cell bodies. Images were acquired using a Nikon TiE 300 inverted microscope (Nikon). A 10× objective was used to create a 3 × 3 stitched image using Nikon Elements Advanced Research software (Nikon). A Texas Red filter was used for FR labeled tissue and green fluorescent protein (GFP) filter used for GFP viral labeled tissue. Following acquisition, all images were converted to grayscale and manually counted by a single blinded individual. Lamina counts were performed by overlaying segmental lamina maps (Watson et al., 2009) onto each of the tissue sections in Illustrator (Adobe).

Fluoro-Ruby injected at C5–6 has the potential to retrogradely label any neuron with a projection at or near the injection site and we found labeled neurons at all spinal levels that were cryosectioned (L1–4). However, the rostrocaudal spread of labeled neurons at L2–3 in both viral labeled groups is limited by the rostrocaudal spread of the virus at the injection site(s) and the need for dual viral transfection to confer labeling. For equal comparison between groups, the number of quantified sections in the FR group was limited to 61 tissue sections per animal, the average number of sections quantified in the viral labeled groups. Additionally, one animal in the HiRet group was excluded from analyses for total and relative number of labeled neurons as the rostrocaudal spread of labeled neurons was half of that seen in all other virally labeled animals; this is likely attributable to an inaccurate or missed intraspinal injection.

### MATLAB Application Development, Quantification, and Validation

A custom application was built using MATLAB and incorporates the image processing techniques color thresholding and boundary determination to determine the number of labeled cells within a user-specified region of interest in the spinal cord. The specific functions used to build the application can be found in Supplemental Document 1. Of the cell detection methods utilized in the MATLAB program, the elimination of background involves pixel-based thresholding, while cell counting is a combination of pixel and object-based detection based on image properties. First, the background of the image is eliminated using thresholding by labeling color and pixel value. Cells are then detected and counted based on the image region properties of pixel area and eccentricity. Pixel area is defined as the actual number of pixels within a region, while eccentricity is the ratio of the distance between the foci of the ellipse and its major axis length for each object region. The application was integrated into an interactive application and graphical user interface (GUI) created with MATLAB’s App Designer. The GUI enables users to seamlessly navigate through a large number of images, while the semi-automated cell counting function eliminates variability between users and reduces quantification time. The application also allows user to easily overlay anatomical maps or grids for more detailed quantification. To validate the accuracy of the MATLAB application, the number of labeled somata counted using the application was compared to the number of somata counted manually and the correlation between the counting methods assessed. The application has been uploaded to an online data repository and can be accessed using this source code/DOI link for public use^3^. The instructional video^4^ provides a step-by-step tutorial for the program, and written instructions are in Supplemental Document 1.

### Statistical Analyses

Results for the total and relative number of somata labeled between groups were compared using an analysis of variance followed by Tukey HSD *post hoc t*-tests using SPSS version 22 (IBM). Results for the percent of labeled somata by lamina were compared using a multivariate analysis of variance followed by Tukey HDS *post hoc t*-tests where appropriate in SPSS. *P* values for all analysis were considered statistically significant when *p* ≤ 0.05, and two-tailed *p* values are reported for *post hoc t*-tests. Pearson correlation was performed to evaluate the relationship between counting methods using RStudio version 1.2.5042. Results for the differences between counting methods by group were assessed using a one-way analysis of variance followed by a Tukey HSD *post hoc t*-test using SPSS.

## Results

### Tracing Methods Have Different Efficiencies

**FIGURE 2:**
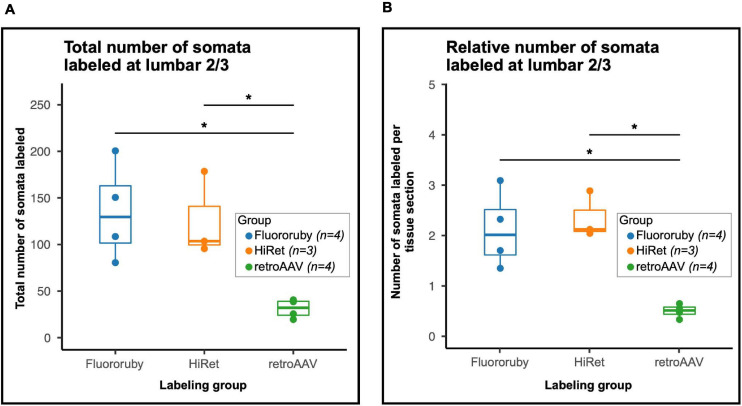
The number of labeled neurons in the lumbar spinal cord is significantly impacted by the tracing method(s) used. **(A)** The total number of labeled neurons differed between groups [One-way Analysis of Variance (ANOVA), *F* = 13.5, df = 2.8, *p* = 0.003] and was lower in the retroAAV group [Tukey’s Honest Significant Difference (HSD) *post hoc*, FR vs. retroAAV, *p* = 0.015; HiRet vs. retroAAV, *p* = 0.034]. **(B)** When normalized to the number of tissue sections counted, the number of labeled neurons differed between groups (One-way ANOVA, *F* = 8.6, df = 2.8, *p* = 0.01) and was significantly lower in the retroAAV group (Tukey’s HSD *post hoc*, FR vs. retroAAV, *p* = 0.006; HiRet vs. retroAAV *p* = 0.005). Panels **(A,B)** are Tukey style box plots. Bold center line shows median, upper hinge shows 75th percentile, lower hinge shows 25th percentile, whiskers represent 1.5 times the interquartile range. Individual data points shown for clarity (*p* < 0.05*, ANOVA and Tukey’s HSD *post hoc t*-tests).

To understand the differences in efficiency between tracing methods, we evaluated the total number of neurons labeled and the number of neurons labeled per tissue section. The total number of labeled neurons (mean ± SD; FR: 135.5 ± 52.29, HiRet: 126.33 ± 45.79, retroAAV: 31.5 ± 10.15) was significantly higher in the FR and HiRet groups compared to the retroAAV group (Figure 2A). The relative number of labeled neurons was evaluated by normalizing the number of labeled neurons to the number of sections counted (mean of labeled neurons per section ± SD; FR: 2.12 ± 0.76, HiRet: 2.35 ± 0.47, retroAAV: 0.50 ± 0.13). After normalization, the same differences between groups were seen, with significantly fewer labeled cells in the retroAAV group compared to the FR and HiRet groups (Figure 2B). These results indicate that tracing LAPNs with FR or target-defined projection labeling utilizing HiRet lentivirus provide robust labeling of LAPNs, while target-defined projection labeling using retroAAV significantly reduced labeling of LAPNs. Additionally, as seen in Figures 1B,D,F, the prominence and detail of labeled neurons differed between FR and virally labeled neurons, with viral labeled neurons often being brighter and easier to identify. However, signal from both viral and FR labeled neurons can be amplified using immunohistochemistry and labeling with a lower molecular weight dextran amine can further improve signal as these dextran amines are more efficiently trafficked (Jiang et al., 1993).

### Specificity of Labeling Is Influenced by Tracing Methods

**FIGURE 3:**
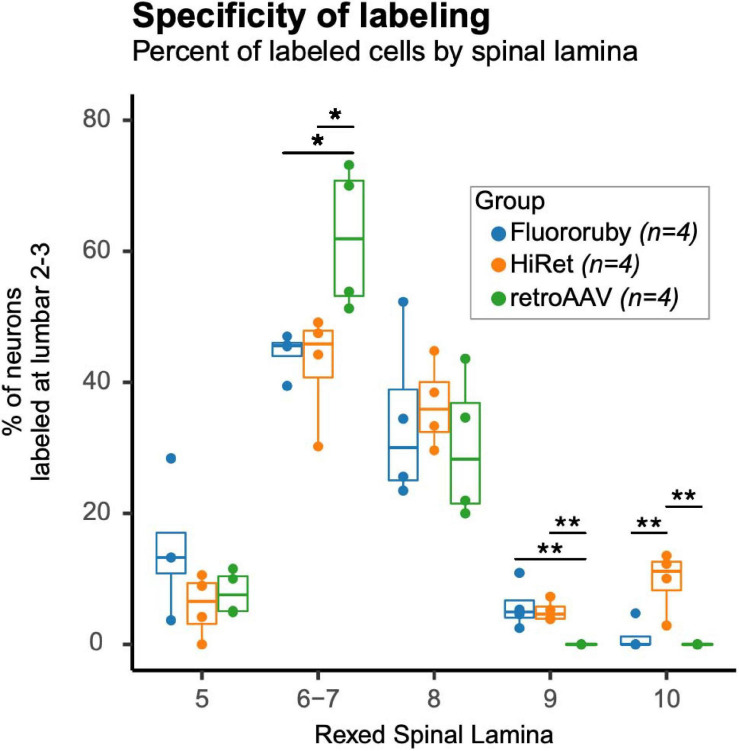
Specificity of labeling is impacted by the tracing method used. Percentages of labeled neurons in Rexed spinal lamina differed between lamina and groups. The retroAAV group had a significantly higher percentage of labeled neurons in lamina 6–7 [Multivariate Analysis of Variance (MANOVA), *F* = 6.6, df = 2.9, *p* = 0.017; Tukey’s HSD *post hoc*, retroAAV vs. FR, *p* = 0.037; retroAAV vs. HiRet, *p* = 0.024] and a significantly lower percentage of labeled neurons in lamina 9 (MANOVA, *F* = 7.7, df = 2.9, *p* = 0.01; Tukey’s HSD *post hoc*, retroAAV vs. FR, *p* = 0.014; retroAAV vs. HiRet, *p* = 0.028). The HiRet group had a significantly higher percentage of labeled neurons in lamina 10 (MANOVA, *F* = 7.7, df = 2.9, *p* = 0.01; Tukey’s HSD *post hoc*, retroAAV vs. FR, *p* = 0.014; retroAAV vs. HiRet, *p* = 0.028). Tukey style box plot. Bold center line shows median, upper hinge shows 75th percentile, lower hinge shows 25th percentile, whiskers represent 1.5 times the interquartile range. Individual data points are shown for clarity. Data points falling outside of whiskers are outlying points that are < *or* > 1.5 times the interquartile range (*p* < 0.05*, *p* < 0.01**, MANOVA and Tukey’s HSD *post hoc t*-tests). Mean percentage ± standard deviation for each lamina and group (FR, HiRet, and retroAAV): lamina 5 (14.6 ± 10.2, 5.9 ± 4.8, 7.9 ± 3.4), lamina 6–7 (44.4 ± 3.4, 42.8 ± 8.6, 62.1 ± 11.1), lamina 8 (33.9 ± 13.1, 36.6 ± 6.6, 30.0 ± 11.1), lamina 9 (5.8 ± 3.6, 5.1 ± 1.6, 0.0 ± 0.0), and lamina 10 (1.2 ± 2.4, 9.7 ± 4.8, 0.0 ± 0.0).

To evaluate differences in specificity of tracing and to determine whether tracing methods preferentially labeled a subset of ipsilateral LAPNs the laminar distribution of the labeled cells was assessed by comparing the percentage of labeled neurons in each lamina (Figure 3). For the absolute number of labeled neurons by animal and lamina see Supplementary Table 1. In lamina 6–7, a greater percentage of neurons was found in the retroAAV group. The retroAAV group also had significantly fewer neurons labeled in lamina 9. In lamina 10, the percentage of neurons was significantly higher in the HiRet group compared to the other groups. These differences in laminar distribution indicate that tracing methods can impact the specificity of labeling either by preferentially targeting a subset of the neuronal population of interest or by random chance.

### Validation of MATLAB Application

**FIGURE 4:**
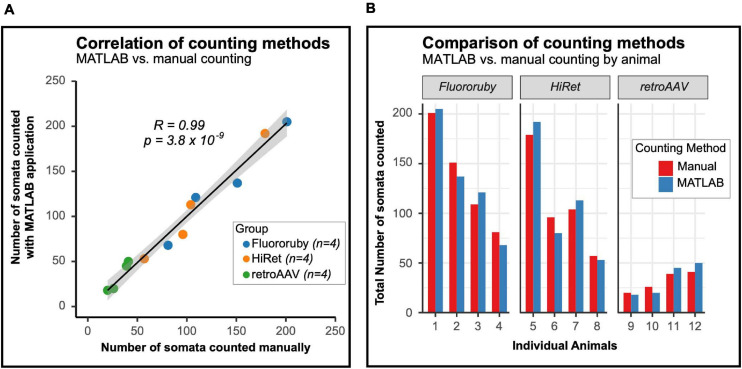
The number of labeled neurons counted with the MATLAB program and manual counting are similar and correlated. **(A)** The number of neurons counted is highly correlated between the MATLAB program and manual counting. Black line indicates line of best fit, gray outline indicates 95% confidence interval (Pearson correlation: *R* = 0.99 and *p* = 3.8 × 10^–9^). **(B)** Animal-by-animal comparison of the number of labeled neurons in the lumbar spinal cord, the difference between counting methods was similar between groups (one-way ANOVA, *F* = 1.7, df = 2.9, *p* = 0.247).

To validate the MATLAB application for quantifying labeled neurons in the spinal cord, the correlation between the number of cells counted manually and by the MATLAB application was assessed. Cell counts using either method were highly correlated with one another (Figure 4A), indicating that the MATLAB application is as accurate as manual counting.

As previously noted, the prominence and detail of labeled neurons differed between FR labeled and virally labeled neurons, with viral labeled neurons being brighter. The poor cellular filling of FR labeled LAPNs may make it difficult for the MATLAB application to detect these neurons. To evaluate potential errors of the MATLAB application in identifying chemically versus virally labeled neurons, absolute differences in the number of neurons counted using each method were calculated and compared between the groups (mean difference scores ± SD; FR: 10.75 ± 4.67, HiRet: 10.50 ± 5.20, retroAAV: 5.75 ± 2.87). These differences were not significant between groups and are visualized animal-by-animal in Figure 4B. Taken together, these results show that the MATLAB application is as accurate as manual counting for quantifying labeled propriospinal neurons and that the application reliably identifies both virally- and chemically-labeled neurons.

## Discussion

### Efficiency of Labeling Methods

Traditional tracers, such as CTB, Fluoro-Gold, and various dextran amines have been extensively characterized in multiple species for tract-tracing throughout the CNS (Lanciego and Wouterlood, 2011; Nassi et al., 2015). However, each of these tracers comes with its own caveats. Dextran amines may inadvertently label damaged fibers of passage (Glover et al., 1986), Fluoro-Gold is neurotoxic (Naumann et al., 2000), and Fluoro-Gold and CTB can label fibers of passage (Dado et al., 1990; Chen and Aston-Jones, 1995). To circumvent these potential limitations and gain a better understanding of viral tropism in the spinal cord, we targeted left ipsilateral LAPNs using target-defined projection labeling (Zeng, 2018). For cell body transduction, AAV2 was chosen over other serotypes as: (1) AAV2 has high neuronal tropism (Bartlett et al., 1998; Burger et al., 2004; Srivastava, 2016), (2) AAV2 has minimal volumetric spread at the injection site (Burger et al., 2004; Aschauer et al., 2013), (3) unlike other serotypes, AAV2 has little potential for retrograde transduction (Hollis et al., 2008; Klaw et al., 2013; Salegio et al., 2013), and (4) AAV2 does not spread transsynaptically like AAV1 and AAV9 (Zingg et al., 2017). These AAV2 characteristics allow precise targeting of the neuronal cell bodies at the injection site. For retrograde transduction and Cre delivery, we utilized the retroAAV serotype developed by Tervo et al. (2016) and the HiRet lentiviral vector developed by Kato et al. (2011b). While we found similar numbers of labeled neurons at L2–3 in the FR and HiRet groups, the retroAAV group showed a 76% decrease in the number of labeled neurons. This is somewhat surprising, as Cre activity is catalytic (Santoro and Schultz, 2002; Gibb et al., 2010). Thus, little Cre expression is needed for FLEx-switch recombination, subsequent transgene expression, and neuronal labeling. One potential explanation for these findings is poor infectivity of LAPN axon terminals. The retroAAV serotype was developed via directed evolution for optimal retrograde transduction of mouse corticopontine neurons (Tervo et al., 2016), but its retrograde transduction of rat LAPNs was modest. RetroAAV has also shown preferential tropism for layer 5 of the cortex when compared to other viral tracers (Sun et al., 2019). As the receptor/co-receptor for this serotype are currently unknown, this modest labeling may result from poor viral uptake at LAPN axon terminals due to little expression of the requisite receptor and/or co-receptor for internalization of retroAAV virions.

Previous studies have shown that changes to the AAV capsid impact the rate of viral degradation and subsequent transgene expression (Zhong et al., 2008a, b; Kay et al., 2013). It is possible that a similar phenomenon is responsible for the poor labeling in the retroAAV group here, as the VP1 region of the AAV2 capsid was altered to produce the retroAAV (Tervo et al., 2016). These mutations may result in increased phosphorylation of viral particles, subsequent ubiquitination, and proteasomal degradation of retroAAV in propriospinal neurons (Zhong et al., 2007, 2008a; Buning and Srivastava, 2019). Lastly, the poor labeling in the retroAAV group may reflect a length-dependent issue, as rat LAPN axons are 6.2–7.6 cm long (Waibl, 2013), approximately 10 times longer than the mouse corticopontine axons (6–7 mm) the retroAAV was developed to target (Oh et al., 2014; Tervo et al., 2016). This may result in poor retrograde trafficking of endosomes containing retroAAV virions. However, Weiss et al. (2020) recently showed successful retrograde transduction of numerous cortical regions in rhesus macaque following intra-caudate and intra-putamen injections. Therefore, we do not believe the poor labeling by retroAAV here is a length-dependent issue. Rather, that this is due to poor uptake and infectivity of retroAAV at propriospinal axon terminals, which may reflect little to no expression of the receptor/co-receptor needed for the retroAAV serotype binding at propriospinal axon terminals.

### Specificity of Labeling Methods

The current data corroborate previous findings that LAPNs in the rat are positioned throughout the intermediate gray matter of the lumbar spinal cord, with the majority residing in laminae 6–8 (Dutton et al., 2006; Reed et al., 2009; Pocratsky et al., 2020). This finding was consistent irrespective of the tracing method utilized. We found a higher percentage of labeled neurons in lamina 10 in the HiRet group. Previous studies found that 12.6% of L2–3 LAPNs reside in lamina 10 (Pocratsky et al., 2020), and that large number of LAPNs are located in lamina 10 (Dutton et al., 2006). The higher percentage seen in the HiRet group here likely reflects the superior retrograde transduction efficiency of HiRet vectors and its ability to efficiently infect LAPN axon terminals compared to retroAAV. Additionally, in the retroAAV group, there was a greater percentage of labeled neurons in lamina 6–7 and fewer in lamina 9 compared to the other groups. These differences reflect either preferential retrograde transduction–or lack thereof–of LAPN sub-populations or are due to a small number of neurons in any lamina resulting in a large percentage change in this group. LAPNs are a heterogenous population of neurons that project ipsi- and contra-laterally (Reed et al., 2006; Pocratsky et al., 2020), have both excitatory and inhibitory neurotransmitter phenotypes (Ruder et al., 2016; Pocratsky et al., 2020), and have varied soma sizes (unpublished data). Future studies may evaluate the neurotransmitter phenotype and soma size of neurons labeled by these tracing methods to determine if differences in the laminar distributions are due to preferential labeling or random chance.

We previously used CTB to retrogradely label LAPNs and found a significantly higher percentage of labeled LAPNs in lamina 5 compared to target-defined projecting labeling with a HiRet vector (Pocratsky et al., 2020). As CTB can be taken up by fibers of passage (Chen and Aston-Jones, 1995), we attributed this difference to inadvertent labeling of lumbar spinocerebellar neurons, a majority of which reside in lumbar lamina 5 (Matsushita and Hosoya, 1979). While we do not report the same finding with FR here, it is important to note that the uptake of dextran amines–such as FR–by damaged axons is more efficient than its uptake by axon terminals (Glover et al., 1986). Thus, when injecting dextran amines, the procedures should minimize damage to the tissue that may occur from osmotic or mechanical pressures.

### Validation of MATLAB Application

Our MATLAB application has semi-automated the quantification of spinal cord labeling, which reduces time, minimizes human error, and allows for anatomical diagrams, such as spinal cord Rexed laminae maps to be easily overlaid on tissue sections for further anatomic characterization. The strong correlation between manual cell counts and those from the MATLAB application (Figure 4A), in conjunction with there being no difference in the error between counting methods for all labeling methods (Figure 4B), emphasizes the accuracy of the MATLAB application for various tracing methods. The detection and counting methods employed in the MATLAB application are effective for small populations of labeled neurons whose boundaries are well defined such as LAPNs, it may not be as effective for larger neuronal populations with more densely packed cells. To accurately detect densely packed cells more advanced object-based detection methods such as edge detection and watershed algorithms might be needed. The program also only extracts either red or green color channels, and if multiple channels are to be detected the images would have to be analyzed twice with each color being counted separately. The application is freely available^5^ and aims to provide a user-friendly application that allows for easy navigation through large numbers of images and the option to overlay anatomical diagrams for further analysis.

Target-defined projection labeling utilizing HiRet vectors showed improved retrograde transduction efficiency compared to retroAAV in the population of propriospinal neurons studied here. This provides the framework for more advanced mesoscale connectomics. This target-defined dual viral approach might also be adapted for exogenous gene expression for therapeutics targeting an anatomically defined set of propriospinal neurons. For these therapeutic approaches to be viable the immune response reported when using lentiviral HiRet vectors must be mitigated (Tanabe et al., 2019). Tanabe et al. (2019) found that lentiviral NeuRet vectors which utilize the fusion E glycoprotein, rather than the fusion B2 glycoprotein used in HiRet vectors, produced no immune response in the primate brain. However, others have reported poor retrograde transduction efficiency of NeuRet vectors when targeting hindbrain and spinal motoneurons (Hirano et al., 2013). Prior to use as a therapeutic for targeting anatomically defined neuronal populations, the retrograde transduction efficiency and immune response of the vectors used should be evaluated. Collectively, the current findings emphasize the need to empirically evaluate and optimize the transduction efficiency of viral vectors and their respective transport properties to target specific neuron population(s).

## Data Availability Statement

The raw data supporting the conclusions of this article will be made available by the authors, without undue reservation.

## Ethics Statement

The animal study was reviewed and approved by University of Louisville Institutional Animal Care and Use Committee.

## Author Contributions

BB, RZ, and CS were responsible for project conception. BB, RZ, CS, and RH performed the experiments. BB wrote the manuscript. RZ and JK developed MATLAB application. DM and SW provided oversight of all experiments and analyses as well as funding. All authors read, revised, and approved the final manuscript.

## Conflict of Interest

The authors declare that the research was conducted in the absence of any commercial or financial relationships that could be construed as a potential conflict of interest.

## Footnotes

1 https://github.com/rachz18/CellCountingApplication.git

2 https://vimeo.com/485620502

3 https://github.com/rachz18/CellCountingApplication.git

4 https://vimeo.com/485620502

5 https://github.com/rachz18/CellCountingApplication.git

## Abbreviations

CNS: central nervous system
AAV: adeno-associated virus
LAPN: long ascending propriospinal neuron
FR: Fluoro-Ruby
C5–6: cervical spinal cord levels 5–6
L2–3: lumbar spinal cord levels 2–3
RDA: rhodamine dextran amine
BDA: biotinylated dextran amine.

## References

[B1] Aschauer D. F. Kreuz S. Rumpel S. (2013). Analysis of transduction efficiency, tropism and axonal transport of AAV serotypes 1, 2, 5, 6, 8 and 9 in the mouse brain. *PLoS One* 8:e76310. 10.1371/journal.pone.0076310 24086725 PMC3785459

[B2] Atasoy D. Aponte Y. Su H. H. Sternson S. M. (2008). A FLEX switch targets Channelrhodopsin-2 to multiple cell types for imaging and long-range circuit mapping. *J. Neurosci.* 28 7025–7030. 10.1523/JNEUROSCI.1954-08.2008 18614669 PMC2593125

[B3] Bartlett J. S. Samulski R. J. McCown T. J. (1998). Selective and rapid uptake of adeno-associated virus type 2 in brain. *Hum. Gene Ther.* 9 1181–1186. 10.1089/hum.1998.9.8-1181 9625257

[B4] Buning H. Srivastava A. (2019). Capsid modifications for targeting and improving the efficacy of AAV vectors. *Mol. Ther. Methods Clin. Dev.* 12 248–265. 10.1016/j.omtm.2019.01.008 30815511 PMC6378346

[B5] Burger C. Gorbatyuk O. S. Velardo M. J. Peden C. S. Williams P. Zolotukhin S. (2004). Recombinant AAV viral vectors pseudotyped with viral capsids from serotypes 1, 2, and 5 display differential efficiency and cell tropism after delivery to different regions of the central nervous system. *Mol. Ther.* 10 302–317. 10.1016/j.ymthe.2004.05.024 15294177

[B6] Cao J. Khan B. Hervey N. Tian F. Delgado M. R. Clegg N. J. (2015). Evaluation of cortical plasticity in children with cerebral palsy undergoing constraint-induced movement therapy based on functional near-infrared spectroscopy. *J. Biomed. Opt.* 20:046009. 10.1117/1.JBO.20.4.046009 PMC4479242 25900145

[B7] Chamberlin N. L. Du B. de Lacalle S. Saper C. B. (1998). Recombinant adeno-associated virus vector: use for transgene expression and anterograde tract tracing in the CNS. *Brain Res.* 793 169–175. 10.1016/s0006-8993(98)00169-3 9630611 PMC4961038

[B8] Chan K. Y. Jang M. J. Yoo B. B. Greenbaum A. Ravi N. Wu W. L. (2017). Engineered AAVs for efficient noninvasive gene delivery to the central and peripheral nervous systems. *Nat. Neurosci.* 20 1172–1179. 10.1038/nn.4593 28671695 PMC5529245

[B9] Chen S. Aston-Jones G. (1995). Evidence that cholera toxin B subunit (CTb) can be avidly taken up and transported by fibers of passage. *Brain Res.* 674 107–111. 10.1016/0006-8993(95)00020-q 7773677

[B10] Dado R. J. Burstein R. Cliffer K. D. Giesler G. J. Jr. (1990). Evidence that Fluoro-Gold can be transported avidly through fibers of passage. *Brain Res.* 533 329–333. 10.1016/0006-8993(90)91358-n 1705157

[B11] Deng L. Ruan Y. Chen C. Frye C. C. Xiong W. Jin X. (2016). Characterization of dendritic morphology and neurotransmitter phenotype of thoracic descending propriospinal neurons after complete spinal cord transection and GDNF treatment. *Exp. Neurol.* 277 103–114. 10.1016/j.expneurol.2015.12.018 26730519 PMC4761305

[B12] Dutton R. C. Carstens M. I. Antognini J. F. Carstens E. (2006). Long ascending propriospinal projections from lumbosacral to upper cervical spinal cord in the rat. *Brain Res.* 1119 76–85. 10.1016/j.brainres.2006.08.063 16996042

[B13] Fox M. D. Raichle M. E. (2007). Spontaneous fluctuations in brain activity observed with functional magnetic resonance imaging. *Nat. Rev. Neurosci.* 8 700–711. 10.1038/nrn2201 17704812

[B14] Gibb B. Gupta K. Ghosh K. Sharp R. Chen J. Van Duyne G. D. (2010). Requirements for catalysis in the Cre recombinase active site. *Nucleic Acids Res.* 38 5817–5832. 10.1093/nar/gkq384 20462863 PMC2943603

[B15] Giovanelli Barilari M. Kuypers H. G. (1969). Propriospinal fibers interconnecting the spinal enlargements in the cat. *Brain Res.* 14 321–330. 10.1016/0006-8993(69)90113-9 5794910

[B16] Glover J. C. Petursdottir G. Jansen J. K. (1986). Fluorescent dextran-amines used as axonal tracers in the nervous system of the chicken embryo. *J. Neurosci. Methods* 18 243–254. 10.1016/0165-0270(86)90011-7 2432362

[B17] Hirano M. Kato S. Kobayashi K. Okada T. Yaginuma H. Kobayashi K. (2013). Highly efficient retrograde gene transfer into motor neurons by a lentiviral vector pseudotyped with fusion glycoprotein. *PLoS One* 8:e75896. 10.1371/journal.pone.0075896 24086660 PMC3782444

[B18] Hollis E. R. II Kadoya K. Hirsch M. Samulski R. J. Tuszynski M. H. (2008). Efficient retrograde neuronal transduction utilizing self-complementary AAV1. *Mol. Ther.* 16 296–301. 10.1038/sj.mt.6300367 18223548

[B19] Horn A. Fox M. D. (2020). Opportunities of connectomic neuromodulation. *Neuroimage* 221:117180. 10.1016/j.neuroimage.2020.117180 32702488 PMC7847552

[B20] Horn A. Reich M. Vorwerk J. Li N. Wenzel G. Fang Q. (2017). Connectivity predicts deep brain stimulation outcome in Parkinson disease. *Ann. Neurol.* 82 67–78. 10.1002/ana.24974 28586141 PMC5880678

[B21] Hu Z. Liu G. Dong Q. Niu H. (2020). Applications of resting-state fNIRS in the developing brain: a review from the connectome perspective. *Front. Neurosci.* 14:476. 10.3389/fnins.2020.00476 32581671 PMC7284109

[B22] Hutson T. H. Verhaagen J. Yanez-Munoz R. J. Moon L. D. (2012). Corticospinal tract transduction: a comparison of seven adeno-associated viral vector serotypes and a non-integrating lentiviral vector. *Gene Ther.* 19 49–60. 10.1038/gt.2011.71 21562590 PMC3160493

[B23] Jeurissen B. Descoteaux M. Mori S. Leemans A. (2019). Diffusion MRI fiber tractography of the brain. *NMR Biomed.* 32:e3785. 10.1002/nbm.3785 28945294

[B24] Jiang X. Johnson R. R. Burkhalter A. (1993). Visualization of dendritic morphology of cortical projection neurons by retrograde axonal tracing. *J. Neurosci. Methods* 50 45–60. 10.1016/0165-0270(93)90055-v 7506340

[B25] Joutsa J. Horn A. Hsu J. Fox M. D. (2018). Localizing parkinsonism based on focal brain lesions. *Brain* 141 2445–2456. 10.1093/brain/awy161 29982424 PMC6061866

[B26] Kato S. Kobayashi K. Inoue K. Kuramochi M. Okada T. Yaginuma H. (2011a). A lentiviral strategy for highly efficient retrograde gene transfer by pseudotyping with fusion envelope glycoprotein. *Hum. Gene Ther.* 22 197–206. 10.1089/hum.2009.179 20954846

[B27] Kato S. Kuramochi M. Kobayashi K. Fukabori R. Okada K. Uchigashima M. (2011b). Selective neural pathway targeting reveals key roles of thalamostriatal projection in the control of visual discrimination. *J. Neurosci.* 31 17169–17179. 10.1523/JNEUROSCI.4005-11.2011 22114284 PMC6623855

[B28] Kay C. N. Ryals R. C. Aslanidi G. V. Min S. H. Ruan Q. Sun J. (2013). Targeting photoreceptors via intravitreal delivery using novel, capsid-mutated AAV vectors. *PLoS One* 8:e62097. 10.1371/journal.pone.0062097 23637972 PMC3637363

[B29] Kitagawa R. Miyachi S. Hanawa H. Takada M. Shimada T. (2007). Differential characteristics of HIV-based versus SIV-based lentiviral vector systems: gene delivery to neurons and axonal transport of expressed gene. *Neurosci. Res.* 57 550–558. 10.1016/j.neures.2006.12.016 17275114

[B30] Klaw M. C. Xu C. Tom V. J. (2013). Intraspinal AAV injections immediately rostral to a thoracic spinal cord injury site efficiently transduces neurons in spinal cord and brain. *Mol. Ther. Nucleic Acids* 2:e108. 10.1038/mtna.2013.34 23881451 PMC3731889

[B31] Klein R. L. Dayton R. D. Tatom J. B. Henderson K. M. Henning P. P. (2008). AAV8, 9, Rh10, Rh43 vector gene transfer in the rat brain: effects of serotype, promoter and purification method. *Mol. Ther.* 16 89–96. 10.1038/sj.mt.6300331 17955025 PMC2987640

[B32] Klein R. L. Hamby M. E. Gong Y. Hirko A. C. Wang S. Hughes J. A. (2002). Dose and promoter effects of adeno-associated viral vector for green fluorescent protein expression in the rat brain. *Exp. Neurol.* 176 66–74. 12093083 10.1006/exnr.2002.7942

[B33] Lai B. Q. Qiu X. C. Zhang K. Zhang R. Y. Jin H. Li G. (2015). Cholera toxin B subunit shows transneuronal tracing after injection in an injured sciatic nerve. *PLoS One* 10:e0144030. 10.1371/journal.pone.0144030 26640949 PMC4671609

[B34] Lanciego J. L. Wouterlood F. G. (2011). A half century of experimental neuroanatomical tracing. *J. Chem. Neuroanat.* 42 157–183. 10.1016/j.jchemneu.2011.07.001 21782932

[B35] Lanciego J. L. Wouterlood F. G. (2020). Neuroanatomical tract-tracing techniques that did go viral. *Brain Struct. Funct.* 225 1193–1224. 10.1007/s00429-020-02041-6 32062721 PMC7271020

[B36] Liu G. Martins I. H. Chiorini J. A. Davidson B. L. (2005). Adeno-associated virus type 4 (AAV4) targets ependyma and astrocytes in the subventricular zone and RMS. *Gene Ther.* 12 1503–1508. 10.1038/sj.gt.3302554 15944733

[B37] Matsushita M. Hosoya Y. (1979). Cells of origin of the spinocerebellar tract in the rat, studied with the method of retrograde transport of horseradish peroxidase. *Brain Res.* 173 185–200. 10.1016/0006-8993(79)90620-6 90539

[B38] Nassi J. J. Cepko C. L. Born R. T. Beier K. T. (2015). Neuroanatomy goes viral! *Front. Neuroanat.* 9:80. 10.3389/fnana.2015.00080 26190977 PMC4486834

[B39] Naumann T. Hartig W. Frotscher M. (2000). Retrograde tracing with Fluoro-Gold: different methods of tracer detection at the ultrastructural level and neurodegenerative changes of back-filled neurons in long-term studies. *J. Neurosci. Methods* 103 11–21. 10.1016/s0165-0270(00)00292-2 11074092

[B40] Oh S. W. Harris J. A. Ng L. Winslow B. Cain N. Mihalas S. (2014). A mesoscale connectome of the mouse brain. *Nature* 508 207–214. 10.1038/nature13186 24695228 PMC5102064

[B41] Okromelidze L. Tsuboi T. Eisinger R. S. Burns M. R. Charbel M. Rana M. (2020). Functional and structural connectivity patterns associated with clinical outcomes in deep brain stimulation of the globus pallidus internus for generalized Dystonia. *AJNR Am. J. Neuroradiol.* 41 508–514. 10.3174/ajnr.A6429 32054614 PMC7077906

[B42] Pocratsky A. M. Shepard C. T. Morehouse J. R. Burke D. A. Riegler A. S. Hardin J. T. (2020). Long ascending propriospinal neurons provide flexible, context-specific control of interlimb coordination. *Elife* 9:e53565. 10.7554/eLife.53565 32902379 PMC7527236

[B43] Reed W. R. Shum-Siu A. Onifer S. M. Magnuson D. S. (2006). Inter-enlargement pathways in the ventrolateral funiculus of the adult rat spinal cord. *Neuroscience* 142 1195–1207. 10.1016/j.neuroscience.2006.07.017 16938403 PMC3741649

[B44] Reed W. R. Shum-Siu A. Whelan A. Onifer S. M. Magnuson D. S. (2009). Anterograde labeling of ventrolateral funiculus pathways with spinal enlargement connections in the adult rat spinal cord. *Brain Res.* 1302 76–84. 10.1016/j.brainres.2009.09.049 19766612 PMC2783768

[B45] Rosenberg J. B. Sondhi D. Rubin D. G. Monette S. Chen A. Cram S. (2014). Comparative efficacy and safety of multiple routes of direct CNS administration of adeno-associated virus gene transfer vector serotype rh.10 expressing the human arylsulfatase A cDNA to nonhuman primates. *Hum. Gene Ther. Clin. Dev.* 25 164–177. 10.1089/humc.2013.239 25144894 PMC4227442

[B46] Ruder L. Takeoka A. Arber S. (2016). Long-Distance descending spinal neurons ensure quadrupedal locomotor stability. *Neuron* 92 1063–1078. 10.1016/j.neuron.2016.10.032 27866798

[B47] Salegio E. A. Samaranch L. Kells A. P. Mittermeyer G. San Sebastian W. Zhou S. (2013). Axonal transport of adeno-associated viral vectors is serotype-dependent. *Gene Ther.* 20 348–352. 10.1038/gt.2012.27 22418061 PMC3381869

[B48] Santoro S. W. Schultz P. G. (2002). Directed evolution of the site specificity of Cre recombinase. *Proc. Natl. Acad. Sci. U.S.A.* 99 4185–4190. 10.1073/pnas.022039799 11904359 PMC123623

[B49] Srivastava A. (2016). In vivo tissue-tropism of adeno-associated viral vectors. *Curr. Opin. Virol.* 21 75–80. 10.1016/j.coviro.2016.08.003 27596608 PMC5138125

[B50] Sun L. Tang Y. Yan K. Yu J. Zou Y. Xu W. (2019). Differences in neurotropism and neurotoxicity among retrograde viral tracers. *Mol. Neurodegener.* 14:8. 10.1186/s13024-019-0308-6 30736827 PMC6368820

[B51] Tanabe S. Uezono S. Tsuge H. Fujiwara M. Miwa M. Kato S. (2019). A note on retrograde gene transfer efficiency and inflammatory response of lentiviral vectors pseudotyped with FuG-E vs. FuG-B2 glycoproteins. *Sci. Rep.* 9:3567. 10.1038/s41598-019-39535-1 30837514 PMC6400974

[B52] Tervo D. G. Hwang B. Y. Viswanathan S. Gaj T. Lavzin M. Ritola K. D. (2016). A designer AAV variant permits efficient retrograde access to projection neurons. *Neuron* 92 372–382. 10.1016/j.neuron.2016.09.021 27720486 PMC5872824

[B53] Ugolini G. (2020). Viruses in connectomics: viral transneuronal tracers and genetically modified recombinants as neuroscience research tools. *J. Neurosci. Methods* 346:108917. 10.1016/j.jneumeth.2020.108917 32835704

[B54] Waibl H. (2013). *Zur Topographie der Medulla spinalis der Albinoratte (rattus norvegicus)/Contributions to the Topography of the Spinal Cord of the Albino Rat (Rattus norvegicus).* Berlin: Springer-Verlag.

[B55] Wang X. Liu Y. Li X. Zhang Z. Yang H. Zhang Y. (2017). Deconstruction of corticospinal circuits for goal-directed motor skills. *Cell* 171 440–455.e14. 10.1016/j.cell.2017.08.014 28942925 PMC5679421

[B56] Watson C. Paxinos G. Kayalioglu G. (2009). *The Spinal Cord: A Christopher and Dana Reeve Foundation Text and Atlas.* Cambridge, MA: Academic press.

[B57] Weiss A. R. Liguore W. A. Domire J. S. Button D. McBride J. L. (2020). Intra-striatal AAV2.retro administration leads to extensive retrograde transport in the rhesus macaque brain: implications for disease modeling and therapeutic development. *Sci. Rep.* 10:6970. 10.1038/s41598-020-63559-7 32332773 PMC7181773

[B58] Wertz A. Trenholm S. Yonehara K. Hillier D. Raics Z. Leinweber M. (2015). PRESYNAPTIC NETWORKS. Single-cell-initiated monosynaptic tracing reveals layer-specific cortical network modules. *Science* 349 70–74. 10.1126/science.aab1687 26138975

[B59] Wong J. K. Middlebrooks E. H. Grewal S. S. Almeida L. Hess C. W. Okun M. S. (2020). A comprehensive review of brain connectomics and imaging to improve deep brain stimulation outcomes. *Mov. Disord.* 35 741–751. 10.1002/mds.28045 32281147 PMC7556988

[B60] Wouterlood F. G. Bloem B. Mansvelder H. D. Luchicchi A. Deisseroth K. (2014). A fourth generation of neuroanatomical tracing techniques: exploiting the offspring of genetic engineering. *J. Neurosci. Methods* 235 331–348. 10.1016/j.jneumeth.2014.07.021 25107853

[B61] Yeh C. H. Jones D. K. Liang X. Descoteaux M. Connelly A. (2020). Mapping structural connectivity using diffusion MRI: challenges and opportunities. *J. Magn. Reson. Imaging* 10.1002/jmri.27188 32557893 PMC7615246

[B62] Zeng H. (2018). Mesoscale connectomics. *Curr. Opin. Neurobiol.* 50 154–162. 10.1016/j.conb.2018.03.003 29579713 PMC6027632

[B63] Zhang Y. P. Burke D. A. Shields L. B. Chekmenev S. Y. Dincman T. Zhang Y. (2008). Spinal cord contusion based on precise vertebral stabilization and tissue displacement measured by combined assessment to discriminate small functional differences. *J. Neurotrauma* 25 1227–1240. 10.1089/neu.2007.0388 18986224 PMC2756607

[B64] Zhong L. Li B. Jayandharan G. Mah C. S. Govindasamy L. Agbandje-McKenna M. (2008a). Tyrosine-phosphorylation of AAV2 vectors and its consequences on viral intracellular trafficking and transgene expression. *Virology* 381 194–202. 10.1016/j.virol.2008.08.027 18834608 PMC2643069

[B65] Zhong L. Li B. Mah C. S. Govindasamy L. Agbandje-McKenna M. Cooper M. (2008b). Next generation of adeno-associated virus 2 vectors: point mutations in tyrosines lead to high-efficiency transduction at lower doses. *Proc. Natl. Acad. Sci. U.S.A.* 105 7827–7832. 10.1073/pnas.0802866105 18511559 PMC2402387

[B66] Zhong L. Zhao W. Wu J. Li B. Zolotukhin S. Govindasamy L. (2007). A dual role of EGFR protein tyrosine kinase signaling in ubiquitination of AAV2 capsids and viral second-strand DNA synthesis. *Mol. Ther.* 15 1323–1330. 10.1038/sj.mt.6300170 17440440

[B67] Zingg B. Chou X. L. Zhang Z. G. Mesik L. Liang F. Tao H. W. (2017). AAV-Mediated anterograde transsynaptic tagging: mapping corticocollicular input-defined neural pathways for defense behaviors. *Neuron* 93 33–47. 10.1016/j.neuron.2016.11.045 27989459 PMC5538794

