# Supplementary material for: Dual-Viral Transduction Utilizing Highly Efficient Retrograde Lentivirus Improves Labeling of Long Propriospinal Neurons
Source: Front Neuroanat. 2021 Mar 22;15:635921. doi: 10.3389/fnana.2021.635921 (PMC8019739; doi:10.3389/fnana.2021.635921)
Supplement: Supplementary file 2 [file Data_Sheet_1.PDF]

Supplemental document 1

MATLAB Application Development

Overview

MATLAB's image processing and AppDesigner functions were utilized to create a custom application for semi-automatic cell counting. Objectives for successful application development included enabling the user to upload an image of a tissue section, select a ROI, process the image, overlay the anatomic map or grid, and detect/count only labeled cells within the selected ROI. The functionality of the application is further discussed here and can also be observed in the instructional video (https://vimeo.com/485620502), which utilizes an example images from the current study.

The specific functions of the program are listed in **Table 1** with a brief description. Along with various functions, there are properties within the application programming that contain object data. These properties are then stored and called throughout the different functions. The specific program properties and a brief description are included in **Table 2**.

Table 1. List and description of application functions

Application Function	Brief Description
startupFcn	Application runs and is maximized to full screen
BrowseImageButtonPushed	Enables user to select desired image using the file selector, displays selected image within axes and file name in adjacent edit box
TraceROIButtonPushed	Enables user to trace region of interest within selected image, new traced image replaces selected image
ColorThresholdSliderValueChanged	Pixels of selected image are thresholded based upon chosen value, image is converted to binary, thresholded image is displayed in axes above slider
LockThresholdButtonPushed	Binary image data is stored as a property, check box is filled if button is pushed
ManualProcessingButtonPushed	Provides user with option to trace image artifact and eliminate it from thresholded image, check box is filled if button is pushed
SelectLaminaButtonPushed	Enables user to select lamina overlay, displays file name in adjacent edit box
DisplayOverlayButtonPushed	Figure window appears displaying original image with selected lamina overlay, size and coordinates of which are specified by the user
CountButtonPushed	Number of cells is counted, figure window displayed with two axes- original image with lamina overlay on the left, thresholded image with boundaries traced around the counted cells on the right
DirectionsButtonPushed	Figure window appears with directions detailing application use

Table 2. List and description of application properties	
**Application Property**	**Brief Description**
Image	Image data corresponding to image selected by user
full_img	Image object created from display of selected image
ROI	Image data corresponding to traced image
thresholdedImage	Image data corresponding to image after thresholding
full_threshold	Image object created from display of thresholded image
processedROI	Image data corresponding to image after manual processing
overlay	Image data corresponding to selected lamina overlay

Application Functions

Upload image of tissue section

The first interactive function of the application is the 'BrowseImageButtonPushed' function. When "Browse Image" button is clicked, the file selector is displayed allowing the user to select an image the tissue section(s). The file selector enables 8-bit and 16-bit images of file type 'png', 'jpeg', 'bmp,' and 'tif' to be selected. The selected image(s) is/are then displayed within the axes under the label "Original Image," and the image data are stored as the property 'Image.' If a 16-bit image is selected, it is automatically converted to 8-bit before being displayed and further processed. The image object is stored as the property 'full_img.' The name of the file is also displayed in the 'Edit Text' box next to the "Browse Image" button.

ROI selection

The 'TraceROIButtonPushed' function enables the user to select a specific region of interest for cell counting. When the "Trace ROI" button is clicked, a separate figure window with the selected image is displayed. The user can then trace the region of interest in which labeled cells should be counted. Once the ROI is traced, each pixel outside of that outline is programmed to turn black, eliminating extraneous background. The new image with eliminated background is displayed within the separate figure window, and the original image is replaced with the new image. The new traced image data are stored as the property 'ROI.' Next to the "Trace ROI" button, there is a drop-down list used to select the type of labeling being analyzed. Select either 'GFP' and 'FluoroRuby' from the drop-down list depending on the tracer/fluorophore used. This selection will determine how the color thresholding is performed in the next step.

Color thresholding and manual image processing

The uploaded and traced image must be further processed before cells can be detected and counted. The 'ColorThresholdSliderValueChanged' function enables the user to eliminate the remaining background, leaving only the labeled cells. The threshold value can be changed by using the slider bar or manually entering a threshold value in the edit box adjacent to the slider bar. The threshold value can be adjusted by the user as needed.

When the value of the color threshold value is changed, the program first extracts the image pixels within the color channel that correspond to the labeling selected using the drop-down list. The traced image is originally stored as an RGB, or “truecolor” image within the property ‘ROI.’ RGB image data includes *m*-by-*n*-by-3 data array that defines red, green, and blue color components of each individual pixel. If ‘GFP’ is selected, only the pixels within the green channel are extracted. If ‘FluoroRuby’ is selected, only the pixels within the red channel are extracted.

When the pixels are extracted, the RGB image is automatically converted to grayscale, and the values of the pixels are converted based upon the selected threshold value. The threshold value is determined by the position of the slider labeled “Color Threshold.” The color threshold slider values range from 0-255. A pixel value of 0 is equivalent to the color black in a grayscale image, while 255 is equivalent to white. Every pixel value below the threshold value is converted to a pixel value of 0, so that the pixels are converted to black. Finally, the thresholded image is converted to binary so that every pixel with a non-zero value is converted to a value of 1.

Once the optimal threshold has been determined, the “Lock Threshold” button is pushed so that the binary image data is stored within the property ‘thresholdedImage’ and displayed within the second axis placed above the color threshold slider. The image object is stored as the property ‘full_threshold.’ The check box adjacent to the “Lock Threshold” button also becomes filled when the button is clicked. The “Lock Threshold” button also allows the user to choose the same color threshold for multiple images without having to manually re-enter the same value using the edit box or slider bar.

After the traced image has been thresholded and converted to binary, the ‘ManualProcessingButtonPushed’ function can be utilized to remove artifacts in the image that could be mistaken for labeled cells. When the manual processing button is clicked, a separate figure window appears containing the thresholded image. The user can then trace around an artifact, and the pixels inside the outlined region will be converted to black, with a pixel value of 0. The image with the blacked-out region will then replace the thresholded image in the second axis above the color threshold slider. If an image has been manually processed, the image data for the processed image is stored in the property ‘processedROI,’ and the check box adjacent to the manual processing button becomes filled.

<u>Lamina overlay (anatomical map or grid) selection and display</u>
The next function is the ‘SelectLaminaButtonPushed’ function. When the button labeled “Select Lamina” is clicked, the file selector is displayed, allowing the user to select a lamina overlay image. The file selector enables 8-bit images of file type ‘png’, ‘jpeg’, ‘bmp,’ and ‘tif’ to be selected. Once the lamina overlay has been selected, the file name is displayed in the edit field adjacent to the “Select Lamina” button. The image data of the lamina overlay are stored as the property ‘overlay.’ Any lamina image can be uploaded to the program but will only work as an overlay if the background of the image has been removed previously.

The ‘DisplayOverlayButtonPushed’ function enables the user to view the selected lamina (or other anatomical maps/grids) overlay onto the original spinal cord section image. When the “Display Overlay” button is pushed, a figure window appears displaying the original image with a lamina overlay, positioned according to the size and coordinate values specified by the user. Five numeric edit fields on the interface allow the user to specify the “Width” and “Height” of the lamina overlay, the coordinate position of the overlay within the x and y axes, and the rotation of the overlay in degrees by manually inputting values. The width and height correspond to the row and column dimensions of the overlaid image. Once the dimensions and position of the lamina

overlay have been specified, the original image with a lamina overlay is displayed. The size and coordinates of the lamina overlay can be updated, and the image re-displayed as needed.

Detection and counting of labeled cells

The 'CountButtonPushed' function detects and counts labeled cells based on the thresholded binary image. When the "Count" button is pushed, the application is programmed to utilize image region properties and MATLAB's 'ncount' function to generate the number of labeled cells and display that number in the numeric edit field adjacent to the "Count" button. To ensure that only labeled cells are counted, the application is programmed to count the regions that have a pixel area greater than 100 and an eccentricity less than 0.97. Pixel area is the actual number of pixels within a region, while eccentricity is the ratio of the distance between the foci of the ellipse and its major axis length. Eccentricity values range from 0 to 1, 0 being a perfect circle and 1 being a line segment. The eccentricity threshold value was included in the count function so that dendrites without a visible cell body are not miscounted as labeled cells, because dendrite shape resembles a line segment. The threshold values for pixel area and eccentricity were determined after 10 test images from each of the 12 animals were run through the application, and the number and location of counted cells was directly compared to the number and location of manually counted cells for each image.

An *elseif* expression is also programmed within the 'CountButtonPushed' function so that the correct image is analyzed if the manual image processing button has been utilized. If the check box adjacent to the manual processing button is not filled, indicating that the image has not been manually processed, the data stored in the 'thresholdedImage' property is called. If a check appears in the box, indicating an image has been manually processed, the image data stored in the 'processedROI' property will be called and analyzed.

After the number of regions, or cells, has been counted in the image, MATLAB's 'bwboundaries' function is utilized to trace the exterior boundaries of the counted cells. A *for* loop is utilized so that for every region in the image, the properties are called to determine the pixel area and eccentricity, and then the regions that have an area > 100 pixels and an eccentricity < 0.97 are traced with a red boundary outline. A new figure is then displayed with two subplots. The subplot on the right displays the thresholded (or thresholded and manually processed) image with the image regions, or labeled cells, outlined in red. The subplot on the left displays the original spinal section image with the lamina overlay, as is displayed when the "Display Overlay" button is clicked. The user can then zoom in on either subplot to determine which lamina the cells are located.

Lastly, a "Directions" button is located in the bottom left corner of the application. When this button is clicked, a separate pop-up window appears with directions detailing application use for reference.

Access to MATLAB application:

Application has been uploaded to an online data repository and can be accessed using this source code/DOI link for public use: https://github.com/rachz18/CellCountingApplication.git

Link to "How To" video:

A "how to" video detailing application use can be found at: https://vimeo.com/485620502.

Trademark Statement: *MATLAB and Simulink are registered trademarks of The MathWorks, Inc. See mathworks.com/trademarks for a list of additional trademarks.*
